# Dieting Behavior Characterized by Caloric Restriction and Relation to Sleep: A Brief Contemporary Review

**DOI:** 10.3390/ijerph20010276

**Published:** 2022-12-24

**Authors:** Vivian Cao, Alisha Clark, Brooke Aggarwal

**Affiliations:** 1 Touro College of Osteopathic Medicine, New York, NY 10027, USA; 2 Division of Cardiology, Department of Medicine, Columbia University Irving Medical Center, New York, NY 10032, USA

**Keywords:** caloric restriction, sleep, dieting

## Abstract

There is sufficient evidence showing that greater sleep quality improves weight loss outcomes achieved through dietary modifications; however, the effects of dietary modifications such as caloric restriction on sleep outcomes is less established. Caloric restriction is a commonly recommended weight-loss method, yet it may result in short-term weight loss and subsequent weight regain, known as “weight cycling”, which has recently been shown to be associated with both poor sleep and worse cardiovascular health. The purpose of this brief narrative review was to summarize the evidence from recent studies of the effects of caloric restriction on sleep. Six articles were identified that specifically measured effects of a caloric restriction-based intervention on aspects of sleep as primary or secondary outcomes. Most research to date indicates that caloric restriction improves sleep outcomes including sleep quality and sleep onset latency. However, the relation between caloric restriction and sleep duration is less clear. Given the mixed results and the potential for severe caloric restriction to lead to weight cycling, future studies are needed to clarify how caloric restriction affects sleep and the potential implications for weight-management efforts.

## 1. Introduction

During the last century, there has been an increase in the prevalence of obesity in the U.S. population. According to the National Health and Nutrition Examination Survey, the percentage of adults aged 20–74 who have obesity has increased from 13.4% in 1960–1962 to 42.8% in 2017–2018, while the prevalence of severe obesity, defined as a BMI greater than or equal to 40, in this population has also increased from 0.9% to 9.6% [1,2]. Cross sectional data shows a concurrent increase in the prevalence of weight loss attempts in the United States [2]. Current measures also show that sleep duration has significantly decreased among all American adults [3]. Poor sleep behaviors such as insufficient sleep and other lifestyle factors, such as sedentary behavior, are some of the driving mechanisms behind the increased prevalence of obesity [4]. Other poor sleep habits include nocturnal sleep awakenings, waking after sleep onset, prolonged sleep onset latency, and poor sleep efficiency.

Insufficient sleep has been associated with a higher risk for adverse health outcomes such as obesity, diabetes, and cardiovascular disease [5,6,7]. Multiple studies have found associations between inadequate sleep and increased caloric consumption. Hedonic (i.e., reward-driven) factors influence increased caloric restriction (CR) when sleep is restricted, rather than homeostatic (i.e., energy balance-driven) factors [8,9]. This is demonstrated by neural imaging studies that found that sleep restriction increases activation of brain reward and food-sensitive centers [10]. Insufficient sleep also plays a role in hormonal energy balance, causing increased levels of ghrelin, decreased levels of leptin, and increased appetite for energy dense foods [8]. Shorter sleep duration also increases the opportunities for eating throughout the day, which may ultimately lead to increased caloric consumption. Lack of sleep can also increase physiological distress, which can be temporarily alleviated with energy-dense foods [8]. Increased caloric consumption caused by insufficient sleep is one of the many proposed mechanisms behind the rise in obesity prevalence.

CR is a common weight loss strategy that is defined as a decrease in caloric intake below what a person would normally consume [11,12]. It is important to note that CR refers to a deficiency of calories, but not necessarily nutrients [12]. The potential benefits of CR have been previously reviewed. For example, CR may provide cardiovascular benefits and provide protection against development of type 2 diabetes [11,13].

While weight loss interventions are recommended for patients with obesity, many previous studies have documented the difficulty of maintaining long-term weight loss [14,15]. Weight cycling, also commonly referred to as “yo-yo dieting”, is defined as losing and/or gaining 10 lb. or more in one year, and previous studies have shown that weight cycling is associated with adverse health outcomes such as poorer cardiovascular health and poorer sleep quality [16,17,18].

Multiple weight loss attempts may also have psychological implications. Psychiatric disorders, particularly depression, are strongly and bi-directionally associated with obesity and sleep [19,20]. Previous studies have shown that increased dieting frequency is associated with eating disorder symptoms [21,22]. Studies have also found that there is an association between low self-esteem or dissatisfaction with appearance with more frequent dieting [21,23]. Although “dieting behavior” has potential negative psychological effects, successful weight loss attempts can improve psychological outcomes such as depressive symptoms and health-related quality of life [24]. 

Most studies that examine both dieting behavior and sleep investigate how they impact weight loss outcomes. Better sleep quality and longer sleep duration have been shown to increase weight loss success [25]. The primary objective of this brief narrative review was to examine recent research on the impact of dieting behavior, as characterized specifically by CR, and sleep. A secondary objective of this review was to identify research gaps in this area, and discuss relevant public health implications for the prevention and treatment of obesity.

## 2. Materials and Methods

**Figure 1:**
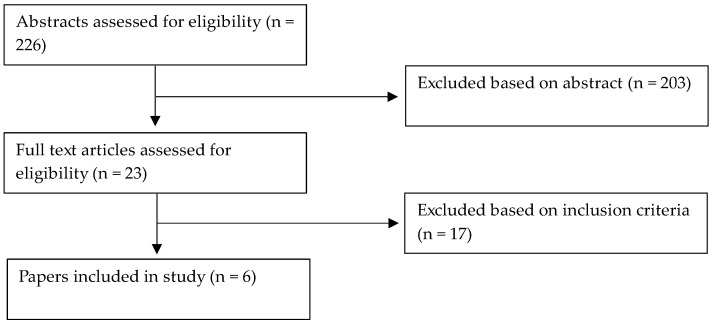
Consort Diagram of the Review Process.

A PubMed search was performed using the following search terms: “caloric restriction”, “restrained eating”, “hypocaloric diet”, “dieting”, “sleep”, “sleep quality”, “sleep duration”, “sleep efficiency”, “insomnia”, “sleep apnea”. The reference sections of the identified articles and Google Scholar were also reviewed for relevant articles. Inclusion criteria for articles included: (1) studies with CR as part of the intervention and sleep measurements as primary or secondary outcomes, (2) randomized control trials (RCT) and non-randomized trials, (3) observational studies. Exclusion criteria for articles included: (1) intermittent fasting and time restricted eating trials, (2) trials with diets related to religion (e.g., Muslim sunnah fasting), (3) investigation of the effect of specific foods in the diet content without CR, (4) manipulation of sleep was part of the intervention. Specific dietary interventions, such as intermittent fasting or a Mediterranean diet, were excluded to examine CR independently from specific nutrient or timing modifications and chronotype, as these has been previous well-documented [26,27]. Only publications written in English between 2002 and August 2022 were considered. A total of 6 studies were included. A diagram of the review process is shown in Figure 1.

### Sleep Outcome Descriptions

The following sleep outcomes were discussed in the articles included in this review. Sleep quality does not have an established definition [28] but often refers to the subjective and objective measurement of an individual’s quantitative sleep measures and includes sleep efficiency, sleep latency, sleep duration, and wake after sleep onset [29,30]. The subjective measurement of sleep quality is frequently determined using the Pittsburgh Sleep Quality Index (PSQI), a validated instrument that measures quality and duration of habitual sleep [31]. It is a self-rated questionnaire that includes 7 components: subjective sleep quality, sleep onset latency, sleep duration, habitual sleep efficiency, sleep disturbances, use of sleep medication, and daytime dysfunction. The lower the sum of all scores, i.e., the global PSQI Score, the higher the reported quality of sleep. The Epworth sleepiness scale (ESS) is used for describing the level of daytime sleepiness in adults [32]. The scores range from 0 to 24 with 0 being no daytime sleepiness and 24 being the highest level of daytime sleepiness. The Basic Nordic sleep questionnaire (BNSQ) consists of 27 items that assess a variety of sleep complaints including subjective sleep quality, and excessive daytime sleepiness. Sleep onset latency (SOL) is defined as the time it takes a person to fall asleep and is measured by wrist worn actigraphy [33] or by an online sleep monitoring system that included a piezoelectric bed sensor that measures sleep/wake status every 30 seconds by analyzing heart rate variability, respiratory rate variability, and binary actogram [34].

## 3. Results

### 3.1. Caloric Restriction and Sleep Outcome 

A limited number of studies examine the direct effects of CR on sleep (Table 1). Four studies were identified in which the intervention was solely CR, and sleep characteristics were a measured outcome. One multi-site RCT (Comprehensive Assessment of Long-term Effects of Reducing Intake of Energy Phase 2 [CALERIE 2]) followed non-obese men (n = 66) and women (n = 152) for 24 months [35]. Participants were randomized into either a 25% CR group or an ad libitum (AL) group. Sleep outcomes were measured in this study using the PSQI. At 12 months, the CR group had improved sleep duration compared to the AL group (Between Group Difference, −0.26; 95% CI, −0.49 to −0.02; Effect Size, −0.32) (*p* = 0.03). However, no significant differences were seen in sleep duration at 24 months. Additionally, the authors found that within the CR group, a greater percent weight loss at month 24 was associated with better sleep quality, according to the total PSQI score (ρ = 0.28; *p* < 0.01).

In contrast, a Canadian cross-over study that included a personalized diet plan targeting 500–700 kcal reduction and a diet based on Canada’s Food Guide to promote a negative energy balance. This study included overweight and obese adult men (n = 83) and women (n = 67) and did not find any significant difference in sleep duration after 12–16 weeks of interventions [36]. However, sleep quality, based on the PSQI, was significantly improved in response to the interventions (*p* < 0.01). In an RCT in a population of patients with Multiple Sclerosis, ages 18–50 years, in which participants (n = 36) followed CR diets. In this study, 7 men and 29 women were randomized to 1 of 3 diets: CR 22% daily reduction, CR 75% reduction twice a week, and control with no reduction in energy needs. There were no significant differences in sleep quality at 8 weeks of intervention (*p* > 0.05) [37].

In a sub study of a clinical trial in overweight individuals (n = 2020) with pre-diabetes, participants who were on a daily energy deficit diet of 800 kcal/day and successfully lost the minimum weight threshold (8% of their baseline body weight) to continue in the study, saw significant improvements in their sleep duration when controlled by group (*p* = 0.0385) and by group and sex (*p* = 0.0443) [38]. There were insignificant improvements in sleep quality when controlled by group (*p* = 0.0508) and when controlled by group and sex (*p* = 0.2136).

### 3.2. Caloric Restriction within Specific Diets and Sleep 

Two studies were found that included CR with an additional dietary modification as the intervention and sleep as the outcome. A Finnish RCT of overweight and obese men (n = 49) with chronic insomnia symptoms found that men on an individualized nutrient-optimized CR diet had shorter objective SOL compared to controls (*p* < 0.001) [34]. Further, total sleep time (*p* = 0.004), SOL (*p* < 0.001), and sleep efficiency (*p* = 0.004) improved within the diet group after the intervention. 

In a RCT with parallel study design of overweight or obese adults (n = 51), participants were randomized into CR diets, restricted by 750 kcal/day, following the Healthy US-Style Eating Pattern with high protein or with the recommended protein quantity [33]. Participants on the CR diet with higher amounts of protein had improved sleep efficiency (1 ± 1%, *p* < 0.027), global sleep scores (−3.8 ± 0.4 au, *p* < 0.001), and daytime sleepiness scores (−3.8 ± 0.4 au, *p* < 0.001) than the CR diet with recommended amounts of protein. However, there were no significant changes found in time spent in bed, time spent sleeping, sleep onset latency, and time after sleep onset between the two groups.

## 4. Discussion

Overall, although results were heterogeneous, there appears to be a positive effect of caloric restriction on most aspects of sleep. Five of the six studies included in this review showed positive effects of CR on most sleep outcomes. The findings on CR’s effect on sleep duration, however, were less consistent. One study showed no effect of CR on sleep.

It is difficult to determine the exact effects that CR has on sleep outcomes as the relationship is known to be bidirectional [39]. Further, in many studies, it is challenging to ascertain whether the results are due solely to the effects of CR or if other factors such as unrecorded modifications to diet quality played a role as well. There is a large amount of current evidence that shows the impact that diet content has on sleep [40,41,42]. In Hudson et.al, the authors are unable to conclude that CR brought about the improvements in sleep efficiency since CR was not the only dietary intervention. Dietary nutrition and energy composition are only some of many factors that influence sleep quality. For example, physical activity has been shown to improve sleep quality and even provide some protection against sleep disordered breathing in obese patients [43,44].

In all of the studies mentioned in this review but one, the patient population included patients with overweight or obesity. Previous studies have described the potential benefits of CR in non-obese individuals as protective against chronic diseases and improvement of psychological wellbeing [45,46]. The relationship between increased adiposity and sleep problems has been well studied. People with obesity report increased incidence of subjective sleep disturbances and also have a higher incidence of sleep disorders such as insomnia, obstructive sleep apnea, and restless leg syndrome [47,48]. The mechanisms underlying the association between dieting and sleep in overweight and obese populations are likely related to the positive effects that weight loss has on sleep (e.g., dietary weight loss can reduce the severity of OSA in obese patients) [49,50]. The benefits shown in both obese and non-obese populations support CR as a beneficial lifestyle modification.

SOL is one of the many symptoms of insomnia with others being nocturnal awakenings and waking up too early. Two of the studies included in this review assessed CR’s effect on SOL with varying results [33,34]. A recent meta-analysis found that people with insomnia are not at increased risk of developing obesity compared to those without insomnia [51]. However, there is evidence that insomnia symptoms, such as SOL, can lead to obesity [47]. One of the proposed mechanisms for how sleep loss is a risk factor for obesity is how the hormones related to appetite regulation, leptin and ghrelin, become unbalanced [52]. The increase in ghrelin and decrease in ghrelin can lead to overeating. 

There are a number of studies that investigate sleep as a moderator of weight loss in CR trials [53,54,55]. These studies found a positive relationship between sleep duration and loss of body fat. However, CR can have negative health implications as well. While calories are restricted, it is important that adequate intake of macronutrients and micronutrients is maintained [12]. Severe CR may also lead to a harmful reduction in fat and muscle mass [13]. It is important to distinguish between CR and eating disorders such as anorexia. A secondary hypothesis of the CALERIE study found that there is no increased risk of developing an eating disorder while following a CR diet [56].

Limitations of this study include a limited number of studies specifically examining the relationship between CR and sleep outcomes, the small sample size of some of the studies, a lack of diversity in some of the subject groups, and the use of subjective and self-reported surveys to assess sleep outcomes introducing potential recall bias in some studies.

As the prevalence of obesity in the United States is increasing and average sleep duration among American adults is decreasing, the findings of this review may have several implications for clinicians who are counseling patients regarding weight loss and sleep [1,2,3]. This may be particularly important in addressing women’s health, as previous studies have shown that women have a higher prevalence of sleep disorders compared to men, including insomnia and shorter sleep duration [57,58]. Maintaining a moderate calorie deficit, while monitoring sleep outcomes such as sleep duration and sleep quality, could be an attainable and sustainable goal for patients who are trying to both lose weight and improve their sleep quality. By accomplishing these goals, patients may also reduce their risk of developing chronic diseases such as cardiovascular disease and diabetes. 

## 5. Conclusions

In conclusion, most research to date indicates that caloric restriction improves sleep outcomes including sleep quality and sleep onset latency. However, the relation between caloric restriction and sleep duration is less clear. Although it is near certain that the relationship between dieting and sleep is bidirectional [39], untangling the specific mechanisms underlying the impact of dieting behavior such as caloric restriction on sleep may provide insight to support better sleep health and enhance healthy weight loss attempts. Given the limited number of recent studies and mixed results, and the potential for severe caloric restriction to lead to weight cycling, future studies are needed to clarify how caloric restriction affects sleep and the potential clinical implications for weight-management efforts. 

## Figures and Tables

**Table 1:** Caloric Restriction and Sleep in Adults.

Reference	Study Design	Sample Size and Baseline Characteristics	Intervention/ Control	Diet Duration	Dietary Intake Intervention	Sleep Outcome Measurement Method	Sleep Outcome Results	Limitations
Filiatrault et al. [36]	Cross over, pooled data from 4 weight-reducing interventions	n = 150, adults (men, women), BMI (average ± SD): 33.3 ± 3.5 kg/m^2^, age (mean ± SD): 38.8 ± 8.6 yr	Intervention: personalized diet plan targeting CR ** in most cases, some participants were on a control diet based on Canada’s food guide to promote a negative energy balance	12–16 weeks	Average 500–700 kcal/day reduction in diet period	PSQI * (measured at baseline and end of intervention)	Sleep quality was significantly improved in response to weight-loss program (*p* < 0.01)	Small sample size, personalized weight loss plans with variable amounts of CR **
Fitzgerald et al. [37]	RCT ^†^	n = 36, adults with MS, BMI > 23	Participants randomized to 1 of 3 diets: (1) daily CR **, (2) intermittent CR **, or (3) weight-stable diet	8 weeks	(1) 78% of calorie needs 7 days/week (2) 25% of calorie needs 2 consecutive days/week, 100% of dietary needs 5 days/week (3) 100% of caloric needs 7 days/week	PSQI *, (measured at baseline and 8 weeks), FAMS * (measured at baseline, 4 weeks, and 8 weeks)	CR ** was not significantly associated with changes in fatigue or sleep quality (*p* > 0.05)	Short duration, reduced generalizability, reliance on self-reports of dietary adherence
Hudson et al. [33]	Randomized, parallel study design	n = 51, adults (mean ± SEM age: 47 ± 1 y, BMI: 32.6 ± 0.5 kg/m^2^)	Controlled USDA Healthy US-Style Eating Pattern and randomly assigned either 5 or 12.5 oz-equivalent (eq)/d of protein foods	12 weeks	750 kcal/day CR **	PSQI *, ESS *, GSS * (measured at baseline, week 6, and week 12)	No change in time spent in bed, time spent sleeping, sleep onset latency, and time awake after sleep onset. Significant improvement in sleep efficiency (1 ± 1%, *p* < 0.027), subjective measures of GSS (−3.8 ± 0.4 au, *p* < 0.001) and daytime sleepiness score (−3.8 ± 0.4 au, *p* < 0.001)	CR ** is not the only dietary intervention, no causal claim made, more females than males, higher attrition rate in high protein group, dietary adherence unknown
Martin et al. [35]	Multisite RCT ^†^	n = 220, Adults (men, women), BMI: 22–28 kg/m^2^	Intervention: CR ** Control: AL ^‡^	2 years	25% CR **	PSQI * (measured at baseline, 12 months, and 24 months)	Intervention: Subjective sleep quality: mean (SE) = 0.11 (0.05), *p* = 0.07 Sleep duration: mean (SE) = 0.06 (0.07), *p* = 0.75 PSQI total score: mean (SE) = 0.24 (0.20), *p* = 0.47 Control: Subjective sleep quality: mean (SE) = 0.12 (0.07), *p* = 0.14 Sleep duration change: mean (SE) = 1.19 (0.09), *p* = 0.08 PSQI total score: mean (SE) = 0.60 (0.26), *p* = 0.04	Composed of mostly white and female, healthy sample. Possible influence on health-related quality of life on sleep due to more study visits in intervention group.
Tan et al. [34]	RCT ^†^	n = 49, adult males with overweight and obesity and chronic complaints of insomnia, average age ± SD (51.8 ± 8.4 yr), average BMI ± SD (30.9 ± 4.8)	Intervention: 300–500 kcal/day less energy intake. Control: AL ^‡^	6 months	300–500 kcal/day CR	BNSQ *, ESS * (measured at baseline and 6 months)	Between group: Diet group has a shorter objective sleep onset latency (*p* < 0.001). Within diet group: prolonged total time asleep (*p* = 0.004), shorter sleep onset latency (*p* < 0.001), increased sleep efficiency (*p* = 0.004), fewer subjective nocturnal awakenings (*p* = 0.035), and decreased number of nocturia (*p* = 0.029)	Sleep onset latency was the only time-by-treatment sleep parameter difference, dietary intervention was not strictly adhered to, limited dietary records, included patients with mild sleep apnea
Tremblay et al. [38]	Multisite clinical trial	n = 2020 (138 unsuccessful responder women, 1214 successful responder women, 53 unsuccessful responder men, 615 successful responder men); age 25–70 years; BMI > 25 kg/m^2^; pre-diabetic	Low energy diet	8 weeks	800 kcal/day with macronutrient composition of 15–20, 35–40, 45–50% energy as fat, protein, and carbohydrate, respectively	PSQI * (measured at baseline and 8 weeks)	Between group differences: significant for sleep duration (*p* = 0.0385), sleep quality (*p* = 0.0508)	More women participants than men, no racial data provided, multi-site study may cause between-site differences

* BNSQ: Basic Nordic Sleep Questionnaire; ESS: Epworth Sleepiness Scale; PSQI: Pittsburgh Sleep Quality Index; SOL: Sleep Onset Latency; ISI: Insomnia Severity Index; FAMS: Functional assessment of Multiple Sclerosis; GSS: global sleep score; ** CR: Caloric Restriction; ^†^ RCT: Randomized Control Trial; ^‡^ AD: Ad Libitum.

## Data Availability

Not applicable.
